# Stressors and Information-Seeking by Dialysis and Transplant Patients During COVID-19, Reported on a Telephone Hotline: A Mixed-Methods Study

**DOI:** 10.1016/j.xkme.2022.100479

**Published:** 2022-05-10

**Authors:** Yaquelin A. Arevalo Iraheta, Ariana L. Murillo, Erica W. Ho, Shailesh M. Advani, LaShara Davis, Amanda Faye Lipsey, Mindy Kim, Amy D. Waterman

**Affiliations:** 1 David Geffen School of Medicine, University of California, Los Angeles, Los Angeles, California; 2 Terasaki Institute for Biomedical Innovation, Los Angeles, California; 3 Houston Methodist J.C. Walter Jr. Transplant Center, Houston Methodist, Houston, Texas; 4 Beyond Medicine-Patient Advocacy, Los Angeles, California

**Keywords:** COVID-19, coronavirus pandemic, dialysis patients, mixed-methods, PHQ-4, qualitative, quantitative, semi-structured interview, telephone hotline, transplant patients

## Abstract

**Rationale & Objective:**

In early 2020, we activated a telephone hotline, the coronavirus disease 2019 (COVID-19) Kidney or Transplant Listening and Resource Center, to learn more about the impact of the COVID-19 pandemic on the stress and information-seeking behaviors of dialysis and transplant patients.

**Study Design:**

A mixed-methods study including semi-structured, qualitative interviews probing about emotional, health, and financial challenges experienced and quantitative surveys assessing depression and anxiety levels and information-seeking behaviors.

**Setting & Participants:**

99 participants (28 dialysis patients; 71 transplant patients), varying by race and ethnicity (Hispanic, 25.3%; White, 23.2%; Asian, 24.2%; Black, 24.2%), shared their COVID-19 pandemic experiences and information-seeking behaviors by telephone. Interviews and surveys were conducted from June 17, 2020, to November 24, 2020.

**Analytical Approach:**

Qualitative themes were identified using thematic analysis. Frequencies were calculated to assess levels of depression and anxiety using the Patient Health Questionnaire for Depression and Anxiety and types of information-seeking behaviors.

**Results:**

7 themes and 16 subthemes emerged. Themes of commonly reported stressors include postponing medical visits; decreased accessibility of getting medication; difficulty in receiving up-to-date, patient-focused health information and dialysis supplies; and delays in medical appointments. Other stressors include losses of health insurance and income, and increased vigilance in behaviors to avoid contracting COVID-19. 15 participants had moderate to severe anxiety and depression symptoms and reported more frequent and severe panic attacks after the COVID-19 pandemic. Participants sought emotional support from family, friends, and faith communities. They also commonly obtained information from news media and reported needing more transplant-specific updates about COVID-19, and frequent communication from their kidney and transplant specialists.

**Limitations:**

This convenience sample of individuals willing to share their experiences through a telephone hotline may not generalize to all dialysis and transplant patients; stressors related to the COVID-19 pandemic for these patients continue to change.

**Conclusions:**

As the impact of the pandemic continues, needs-based interventions tailored for the kidney and transplant community, including access to mental health resources, education, and support for care transitions, should continue.

**Plain-Language Summary**

To understand patients’ lived experiences during the coronavirus disease 2019 (COVID-19) pandemic, we created the COVID-19 Kidney and Transplant Listening and Resource Center. We conducted a mixed-methods study including semi-structured qualitative interviews probing emotional, health, and financial challenges and quantitative surveys assessing depression and anxiety levels and information-seeking behaviors. Commonly reported stressors included postponing or delaying medical visits and difficulty receiving medications. Fifteen participants with moderate to severe anxiety and depression reported having more frequent and severe panic attacks. Participants reported needing more transplant-specific updates about COVID-19 and frequent communication from their kidney and transplant specialists. As the impact of the COVID-19 pandemic continues, needs-based interventions tailored for the kidney and transplant community, including access to mental health resources, education, and support for care transitions, should continue.

While the world has been combating the challenges of coronavirus disease 2019 (COVID-19), immunocompromised patients, such as patients with chronic kidney disease (CKD) and solid organ transplants, are even more vulnerable. Due to high rates of medical comorbidities, including obesity, diabetes, and chronic heart disease, CKD patients who contract COVID-19 have poor outcomes.^1^, ^2^, ^3^, ^4^

The impact of the COVID-19 pandemic on mental health nationally has been severe, with depression and anxiety rates tripling overall.^5^ Before the COVID-19 pandemic, depression and anxiety were more prevalent among dialysis patients^6^ compared to the general population, and this trend was exacerbated by the COVID-19 pandemic. In a study conducted in Italy during the COVID-19 pandemic, kidney recipients reported psychological and emotional symptoms, such as anxiety, depression, anger, and fear caused by long periods of isolation and potential contagion.^7^ Another study conducted with organ recipients in China between March and April 2020 found that 30.5% of participants struggled with post-traumatic stress disorder and 13.4% struggled with depression.^8^ This study found that transplant recipients with depression scored significantly lower on all areas of a Quality-of-Life Questionnaire, with reports of worsened physical health, pain, vitality, and emotional states. To truly understand patients’ lived experience during a global pandemic, we created the COVID-19 Kidney and Transplant Listening and Resource Center (KTLRC), a telephone hotline to learn, in real time, about the specific challenges and stressors that dialysis and transplant patients were facing and to disseminate transplant-related education about COVID-19, including mental health resources. Hotlines have previously been shown to help manage health issues, such as anxiety and depression, and to prevent suicides.^9^^,^^10^ Previous research has found that telephone helplines are beneficial to people dealing with suicidal ideations,^11^ because users feel safer asking questions on sensitive issues, such as depression, death, and finance, compared to asking via a web-based approach.^12^

Hotlines continue to be critical sources of information and support during the COVID-19 pandemic, as they can be tailored in a culturally appropriate way to connect spiritually with callers.^13^ Hotlines have also been praised as an ideal form of psychological service delivery during the pandemic, as they do not require face-to-face interaction^14^ and can be tailored to audiences such as front-line workers.^15^ Hotlines have not previously addressed specific needs of the dialysis or transplant communities, particularly during a pandemic. To our knowledge, the KTLRC is the first hotline created specifically for dialysis and transplant patients to help them navigate the COVID-19 pandemic.

After surveying dialysis and transplant patients who contacted the KTLRC, we conducted a study with 3 aims: (1) to determine how health-care changes and fears about getting COVID-19 resulted in health-care management, financial, and emotional challenges; (2) to identify coping strategies and information-seeking behaviors; and (3) to assess opportunities for the health-care system to help patients better cope with COVID-19-related challenges.

## Methods

### Approach

This study employed a mixed-methods strategy including semi-structured interviews and surveys. Semi-structured interviews assessed dialysis and transplant patients’ common questions, fears, and stressors during the COVID-19 pandemic, and surveys assessed dialysis and transplant patients’ depression and anxiety levels, coping strategies, and information-seeking behaviors.

### Sample Population

This study was approved by the University of California, Los Angeles Institutional Review Board (20-000863) under a waiver of documentation of consent. Eligible participants were 18 years of age or older and either dialysis or transplant (kidney, liver, lung, etc) patients. A total of 99 dialysis and transplant patients shared their COVID-19 pandemic experiences by participating in a semi-structured interview. Transplant patients were comprised of 68 kidney, 2 heart, and 2 lung transplant patients. Of these 99 participants, 86 completed the semi-structured interview and a short survey, and 13 participants only completed the interview. Most participants were English-speaking kidney transplant patients from the University of California, Los Angeles Health System. Table 1 shows additional participant characteristics.

**Table 1:** Participants’ Demographic Information

Characteristics	Transplant Patients^a^	Dialysis Patients
N	72	27
Age, mean y	54	55
Gender		
Female	35	12
Male	28	11
Did not respond	9	4
Race		
White	20	3
Black or African American	15	9
Asian	17	7
Other	2	1
Ethnicity		
Hispanic	18	7
Primary language		
English	67	25
Spanish	5	2
Education		
HS, GED, or less	12	5
Some college	11	7
Associate or bachelor’s	24	9
Postgraduate	20	4
Did not respond	5	2
PHQ-4 score		
0-5	58	20
6-12	9	5
Did not respond	5	2

Abbreviations: GED, general equivalency diploma; HS, high school; PHQ-4, Patient Health Questionnaire for Depression and Anxiety.

a Transplant patients were comprised of 68 kidney transplant patients, 2 heart transplant patients, and 2 lung transplant patients.

### Recruitment and Interview Procedures

We created and launched the COVID-19 KTLRC in direct response to the unique and unmet needs of the dialysis and organ transplant community during the COVID-19 pandemic. The KTLRC enabled dialysis and transplant patients to share their questions and concerns with trained staff and provided a better understanding of the challenges dialysis and transplant patients faced during the COVID-19 pandemic. Participants were recruited for the study from June 17, 2020, to November 24, 2020, through social media (ie, Instagram, Facebook) and the transplant center’s electronic medical records. These participants were found by searching relevant hashtags, such as #transplant, #dialysis, and so forth, and by messaging people following the “Explore Transplant” Instagram and Facebook. The transplant center at the University of California, Los Angeles gave us a list of transplant patients in their electronic medical records. The total number of patient phone numbers given to us through the electronic medical record was 1526 (declined to participate: 59; the remaining participants were not able to be reached). Verbal informed consent for participation and audio recording was obtained from all participants. Out of the 99 participants, 86 completed the open-ended interview questions and the quantitative survey, and 13 participants only completed the open-ended interview questions. Interested participants either scheduled a convenient interview time or were called directly. Calls were answered by staff trained in motivational interviewing and empathic communication, who were ready to answer questions about COVID-19 and transplant. They provided educational materials and referrals to additional resources, including mental health and financial resources.

After giving verbal consent, patients answered open-ended interview questions about their COVID-19 pandemic experience, including how they were seeking help and support; additional help they needed; questions about COVID-19; how their health care was being delivered and their telehealth experiences; how they were learning about their health; and suggestions for improvement within the health-care community. A detailed interview guide with survey questions can be found in Item S1.

Participants also completed a quantitative survey assessing their demographic characteristics (eg, gender, race and ethnicity), participant type (eg, dialysis patient, transplant patient), primary language spoken, and education level. They were screened for anxiety and depression using the Patient Health Questionnaire for Depression and Anxiety (PHQ-4).^16^ Participants were asked how often they were bothered by 4 different problems (eg, feeling nervous, anxious, or on edge, etc) over 2 weeks. The categories of psychological distress were none (0-2), mild (3-5), moderate (6-8), and severe (9-12).

Finally, participants selected how they were learning about COVID-19 (eg, from social media, health-care institutions, news sources, patient groups, and/or searching the Internet). Participants reported how much they agreed or disagreed with statements regarding their accessibility to health information (eg, I wanted health information that I did not know how to get). Then they chose the types of resources that would be most helpful to them (eg, educational videos, a website with frequently asked questions, a live webinar, and/or a live chat). They answered questions about their comfort using mobile health technology and telehealth (eg, Using mHealth or Telehealth services would make me very nervous), answering on a 5-point Likert scale with answers ranging from “strongly agree” to “strongly disagree.” Questions about how often participants spent their time finding and understanding health information (eg, how often do you understand information about your health?) were answered on a Likert scale from “never” to “always.”

Interviews lasted between 30-60 minutes and concluded after all interview and survey questions were asked. No repeat interviews were conducted, and no field notes were taken. Participants were not compensated for their time but were given educational resources about COVID-19 tailored for transplant patients and caregivers.

### Analysis

The study data were collected and managed using REDCap tools^17^^,^^18^ hosted at the Terasaki Institute for Biomedical Innovation. Interviews were transcribed and analyzed for themes and patterns by trained qualitative staff using Braun and Clarke thematic analysis.^19^ Transcripts were not returned to the participants. Trained investigators in thematic analysis (YAAI and ALM) began the analysis by identifying all instances where participants expressed any area of stress and the challenges resulting from being an immunocompromised patient during the COVID-19 pandemic. The set of codes emerged inductively, by reading through the transcripts and allowing the data to determine the themes. YAAI and ALM met weekly to modify codes, clarify interpretations, reconcile differences, and revise codes. Coded lines were reviewed for themes and subthemes (Fig S1). Qualitative analysis software Dedoose (v 8.3.35) (SocioCultural Research Consultants [SCRC]) was used to support the analysis. Quantitative data were analyzed using descriptive statistics in Stata (StataCorp LLC) to calculate frequencies and means of critical variables. Percentages were calculated by the mean of the variable of interest divided by the total count of responses; this number was then multiplied by 100. Critical variables included the study population’s demographics, patient’s information-seeking behaviors, experience using telehealth, and so forth. Participants did not provide feedback on the findings.

## Results

A total of 99 participants participated in the KTLRC; 86 participants completed the entire interview with the survey, and 13 only completed the interview portion of the study. Thirty-nine participants were men, while 47 were women. The participants varied in their race and ethnicity: 23 were White, 24 were Black or African American, 24 were Asian, and 25 were Hispanic. Twenty-seven participants were dialysis patients and 72 had received a solid organ donation (68 were kidney transplant recipients, 2 were heart transplant recipients, and 2 were lung transplant recipients). The primary language spoken was English, for 92 participants, while 7 participants spoke Spanish. The level of education among the participants varied: 17 had a high school diploma or general equivalency diploma, 18 had completed some college, 33 had received an Associate’s or Bachelor’s degree, and 24 had completed some postgraduate education.

After being screened for anxiety and depression using the PHQ-4, 15 out of 86 participants reported having moderate to severe anxiety and depression. Patients with a moderate to severe PHQ-4 score reported having increased anxiety and panic attacks compared to those participants with a lower PHQ-4 score.

A quantitative analysis also indicated that participants consumed health information at least 2 times weekly via the Internet (56.57%), television (40.57%), or print sources (31.13%). They were more likely to learn about health issues from news media (80.81%) than health-care institutions (64.45%) (Table 2). Nearly 69% talked about health issues with family or friends twice a week. Participants with high school degrees or less were less likely to agree that the amount of information they had to make health choices was helpful (*P* = 0.002). Generally, participants believed they had the necessary resources (94.62%) and knowledge (93.55%) to access telehealth; however, those with only high school degrees (*P* = 0.002) or Medicare (*P* = 0.80) were less confident. Twenty-nine percent of respondents said they agreed or strongly agreed that they wanted health information but did not know how to get it.

**Table 2:** How Patients Sought Health Information During the COVID-19 Pandemic

How Patients Learned About COVID-19	Percentage	Transplant	Dialysis	*P* Value
From news sources	80 (80.81%)	57 (80.28%)	23 (82.14%)	0.61
From their health-care institutions	64 (64.45%)	46 (64.79%)	18 (64.29%)	0.96
By searching the Internet	53 (53.54%)	41 (57.75%)	12 (42.86%)	0.18
From their friends and family members	46 (46.46%)	34 (47.89%)	12 (42.86%)	0.65
From social media	35 (35.35%)	24 (33.80%)	11 (39.29%)	0.61
From patient groups	12 (12.12%)	9 (12.68%)	3 (10.71%)	0.78

Abbreviation: COVID-19, coronavirus disease 2019.

A qualitative analysis determined that coping with the possibility of contracting COVID-19 and taking preventive actions to avoid it increased transplant and dialysis patients’ emotional, health, and financial stress, especially for those with higher rates of anxiety and depression. Exemplar quotes are provided to demonstrate key themes (Table 3, Table 4, Table 5).

**Table 3:** Emotional Challenges During the COVID-19 Pandemic

Themes	Exemplar Quotes From Participant Interviews	
**Due to fear of contracting COVID-19**		
	PHQ 4 score 0-5	PHQ-4 score 6-12
Fear of contracting COVID-19, because of a weakened immune system.	“I'm afraid of getting the flu. I'm afraid of getting anything … I know my body has a hard time fighting it because it's immunosuppressed. So yes, I'm afraid of getting it and that's why I'm not exposing myself.” - Transplant Patient 9	“… I guess the fear of getting it and, and not overcoming it because of, you know, the health situation that I have.” - Transplant Patient 52
Increased vigilance due to being immunocompromised led to increased anxiety levels.	“Just like when I’m in – when I do have to go into the grocery store. People don't honor the 6 feet social distancing. People are wearing the mask wrong, and it's just like my anxiety just kicked in. That's it.” - Transplant Patient 704	“And at this point, it's just causing an incredible amount of anxiety because school starts in 3 weeks … it really is taking a toll on mental health just constantly trying to be like, how am I going to protect myself?” - Transplant Patient 1022
**Due to actions taken to prevent exposure to COVID-19**		
Staying at home resulting in extreme isolation and anxiety or panic attacks.	“Just, I would say extreme isolation. I live by myself and I'm single. So, I haven't seen, I haven't had interaction with another human really since March 9th" - Transplant Patient 86	“… first time in my life I was ever had a panic anxiety attack at 44 years old after being a Marine. And everything in my life. I've never panicked and had a panic attack until I was in isolation due to COVID.” - Transplant Patient 13
Increased vigilance led to patients having strained relationships with family members.	“It's affecting my family as well. You know my husband because he still has to go to work. And because I'm too cautious and I, I worry and too scared that, he's bringing the virus in, and then might infect me. And I can't let my daughter like I have, you know it's strained my relationship with my daughter because I don't let her go out and then always because I worry that you show–that she goes out and meet her friends and it puts me at risk.” - Transplant patient 52	

Abbreviation: COVID-19, coronavirus disease 2019.

**Table 4:** Health-Care Management Challenges During the COVID-19 Pandemic

Themes	Exemplar Quotes from Participants’ Interviews
**Due to actions taken to prevent exposure to COVID-19**	
Preventing exposure to COVID-19 stopped patients from entering medical and public spaces.	“Staying out of the public realm so as not to catch the sickness, deciding to go into the doctor or stay home and just call, going and get my shots at a place that I can trust. I recently had a terrible experience at CVS waiting for a shingle shot, and it made me walk out without it because their place where they administer the shots was filthy and obviously hadn't been cared for, sanitized, wiped down or anything … I'm just afraid to go back to that drugstore.” – Transplant Patient 25
Preventing exposure to COVID-19 led to postponed medical visits.	“So, I see my transplant center about 3 times a year, 3 to 4 times a year … They wanted to see me and then I was like, ‘No. I’m not coming in.’ And then I was going to push it off again and they’re like, ‘no. You haven’t gotten bloodwork since February. You have to get your blood work done.’ And you have to do a telehealth appointment because that’s like the longest I went without seeing them. Or getting bloodwork done.” - Transplant Patient 15
Preventing exposure to COVID-19 affected accessibility of getting medication.	“… every time my daughter has to go to Rite Aid. She's getting exposed. And she's about as panicked as I am about exposure. So, you know this it's, it's, it's crazy. I'm thinking maybe I'm going to try and do apparently CVS will actually mail the drugs to you.” - Transplant Patient 41
**Due to lack of resources or support from the health-care community**	
Difficulty receiving up-to-date, patient-focused health education or information.	“The reason why I called - the number wasn't to participate in the survey, but rather to get some more information about how to communicate to my doctors and my transplant team that I need some help getting reasonable accommodations. My employer, because I'm a high school teacher, so it's like a very high-risk environment. And I just found out about the survey that way, and it made sense to me that, like, I'm spending, I'm spending all this time trying to get these resources and trying to find out this information. And there are lots of transplant patients out there who are also probably in a similar boat and need, you know, we need to kind of advocate for ourselves” - Transplant Patient 1022
**Challenges receiving health care during COVID-19**	
Difficulty working within telehealth platforms	“I’m kind of forced to do the telehealth because that's the way they would rather do it. But I missed the face-to-face interaction. So, I would hope that someday we can get back to actually sitting face to face with your doctor, because that's a little more, that appeals to me a little more than talking to a computer screen.” - Transplant Patient 430
Delays in appointments and not being able to see their doctor in a timely fashion.	“But I think overall, I think some of it is the fact that, you know, being a transplant recipient and not being able to get to my doctors in a timely fashion, have been a struggle …” - Transplant Patient 2
Delays in getting a kidney transplant.	“And I'm still waiting to hear from the pretransplant unit whether I've been accepted into the new program or not. And when I call, they just say that, ‘We're really backed up because of COVID,’ but nobody's giving me a clear-cut answer. Did you get my referral? Like, am I in the system? Like, I have living donors that are willing and asking me questions, but I can't give them any answers to the questions they're asking because nobody is telling me whether they got the referral and they just say, ‘We're really backlogged.’ Like, we'll get to you eventually.” - Transplant Patient 84
Difficulty receiving dialysis supplies.	“[My dialysis supply company] are not delivering my actual supplies, they're not putting it upstairs in like the room that we originally designated. And it's hard to get like these boxes of fluid upstairs. So, they you know, all today because of COVID … And sometimes I have like, over 20 boxes to get upstairs and it's really hard. You know, to get those–to get these heavy boxes upstairs. So, I just wish, I mean. I mean, I know they're trying to protect their drivers, but I just wish they would find like another alternative.” - Dialysis Patient 19

Abbreviation: COVID-19, coronavirus disease 2019.

**Table 5:** Financial Challenges During the COVID-19 Pandemic

Themes	Exemplar Quotes From Participants’ Interviews
**General financial challenges due to the COVID-19 pandemic**	
Maintaining health insurance due to job losses.	“… so, like with my Medicaid with health insurance, that kind of financial thing because of the cost of my health insurance right now, it is. It's currently my state has a Medicaid program. It's free. But I don't know, it's going to start costing money. And if I don't have a job, how many of you will pay for that? So that's something that's up in the works. It's in the works right now trying to figure out how to keep my coverage. Beyond its coverage only goes until the end of June. I want to kind of know what my options are between June 30. And when I go back to August third the month of July, basically, yeah.” - Transplant Patient 2
Losing employment.	“The loss of the job and not being able to pursue one. I have worked since I was 16 years old. And I have never, even with my transplant, I was out of work for about 5 wk and was back. So, this has been frustrating in that sense.” - Transplant Patient 112
**Due to actions taken to prevent exposure to COVID-19**	
Being unable to work.	“I was an executive chef for a Hilton. I couldn't be at work on advice of my kidney coordinators because it's an international travel.” - Transplant Patient 13 “I cannot go outside to work, because my job is like deliveries, and I have to, like, see a lot of people. And then I have to, you know, take their car to park. And then I have to send, I have to hand my machine to them and bring it back. I cannot do that, you know, because it's really hard to be careful … Yeah. I cannot go work.” - Transplant Patient 88
Being unable to seek home repair support and childcare support.	I wish I could get a handyman in to do all that stuff. But I don't want a guy in. I don't want anybody in my house.” - Transplant Patient 10 “And again, because I have a partner who I live with, and we are coparents, we can split childcare duty, because I can't send my son who's 3 to daycare, even after the daycare is reopened, because, you know, he's already, there's already been a COVID positive at his daycare. I mean, he hasn't been since March. But like childcare is really hard.” - Transplant Patient 1022

Abbreviation: COVID-19, coronavirus disease 2019.

### Emotional and Health Challenges

The emotional and health challenges identified included a fear of contracting COVID-19, increased vigilance, increased anxiety and panic attacks, strained relationships, postponed medical visits, and delayed appointments. For example, 1 transplant patient shared, “I mean, transplant patients, we always have our mortality kind of like in our face, but this is especially bad. I'm back to being locked in and it's very isolating. It's a huge challenge” (Transplant Patient 521). Another transplant patient mentioned how they had to postpone medical visits to protect themselves, saying “I had postponed my actual lab and blood lab until I found that phlebotomist that would come to my home that wasn't going into nursing homes … I'm trying to minimize my risk, I needed to make sure I wasn't bringing that added risk over to my home” (Transplant Patient 9).

Particularly, participants reported becoming more hypervigilant, watching for their behaviors or any behaviors of their family members that might lead to COVID-19 transmission. Several participants mentioned how difficult it was to do essential outings, such as going to the pharmacy or grocery shopping. One transplant patient said, “I get in the market, the minute they open. And all of a sudden, I turn around into an aisle and I looked up and 7 people are clustered around me, and I would just panic” (Transplant Patient 9). A dialysis patient expressed that they consistently felt constrained and vulnerable to the virus: “I can be infected anytime very easy … it’s not safe anywhere you want to go” (Dialysis Patient 610).

After being screened for anxiety and depression using the PHQ-4, 15 participants reported having moderate to severe anxiety and depression. A notable finding in participants with higher PHQ-4 scores was that they reported abnormal anxiety levels and increased panic attacks after the COVID-19 pandemic: a transplant patient mentioned, “I've been having anxiety since I did dialysis, but it wasn't as bad anymore … all of a sudden after the COVID it just started again, times 10” (Transplant Patient 749).

Participants experienced added emotional stressors owing to the actions they took to prevent exposure to COVID-19. Many participants mentioned how their increased vigilance led to having strained relationships with family members. A transplant patient expressed, “I think also when other families are not as, not as careful and diligent about staying socially distancing or wearing masks. I think that puts extra pressure on my son who then feels like he doesn't want to be the only one and then exposure to me putting me at risk” (Transplant Patient 7). One dialysis patient mentioned that isolation affected their entire family, saying, “it’s more stressful, I guess, the quarantine and the isolation. It doesn’t just affect me; it affects my living donor and it affects my children. So, it affects everybody because I need to quarantine, everybody else who lives with me has to do the same” (Dialysis Patient 515).

### Financial Challenges

Financial challenges identified included difficulty maintaining health insurance, losing employment, and the inability to work. When expressing necessary actions taken to prevent exposure to COVID-19, some reported quitting their job, while others decided to not get a job. A transplant patient mentioned challenges finding work due to increased risk, saying “I had been applying for a sales position, and they require me to be out in the public and make calls, but since everything with my immunosuppression, until there's some sort of vaccine, I can't apply for those positions. So, I'm currently on disability” (Transplant Patient 34). Others reported that the COVID-19 pandemic affected their ability to complete home repairs or to have childcare support within the home.

### Coping Strategies and Information-Seeking Behaviors

Participants reported different coping strategies, such as seeking emotional and practical support from family and friends, their faith communities, and the health-care community. A dialysis patient mentioned being supported by their community of faith: “on Sundays we still go to church, but we have like church, on the parking lot and we stay in our cars. So just those things that basically keep my mind off everything” (Dialysis Patient 19). A few participants reported they did not seek or need support from anyone.

A transplant patient mentioned being confused by the data they were getting publicly and on the news: “of you go on a transplant patient web group or on a Facebook page, every transplant center has different recommendations” (Transplant Patient 1022).

Some found it difficult to find reliable information that updated them about the risks of COVID-19, specifically for at-risk patients. For example, a transplant patient shared a concern about “the lack of resources that are available for people with organ transplants. To be honest, it drove me to call [the listening center] because I've been feeling like I've been left in the dark” (Transplant Patient 13). The most common questions concerned their eligibility for and the safety of the COVID-19 vaccine for kidney and transplant patients, recommendations on safe activities during the pandemic, concerns on the effects of COVID-19 on kidney and transplant patients, and their chances of dying if they contracted the virus. A transplant patient asked, “my question is, … since we are transplant patients, would that vaccine … be good for us to try … or should we take it or should we not?” (Transplant Patient 155).

### Stressors Associated With Managing CKD and Dialysis or Transplant Health

Patient-reported stressors working within the kidney health-care system and managing their CKD and dialysis or transplant health included delays in appointments and kidney transplants, as well as difficulty getting dialysis supplies.

### Support Managing CKD and Dialysis or Transplant Health Needed

When asked what the health-care community can do better, patients recommended creating a centralized place to receive COVID-19 updates, providing information for safe social activities, increasing health-care follow-ups, and allowing greater flexibility to choose how they receive health care. A transplant patient mentioned the need for more follow-ups from the health-care community, saying, “I think reaching out via email and saying we’re here for you. If you have unanswered questions, here’s our portal … we don’t want you to feel alone … just that kind of thing, but [the health-care community] need to be touching base with [immunocompromised patients] maybe even monthly” (Transplant Patient 9).

## Discussion

Individuals with advanced CKD, particularly individuals dependent on dialysis and kidney transplant recipients, not only are highly vulnerable to severe complications associated with COVID-19 but also have high prevalences of depression, anxiety, post-traumatic stress, and suicidal ideation,^20^ placing this population at increasingly high risk of developing a mental health crisis during the COVID-19 pandemic. Before the pandemic, some transplant patients were already taking precautions to avoid getting sick, including wearing face masks and being diligent in sanitizing. However, the fear of contracting COVID-19 caused many to report becoming more hypervigilant by restricting their actions within the community to avoid contracting COVID-19 and changing how they managed their health.

This study’s findings from the distress screening cohere with those of studies from other pandemics concerning increased incidences and risks of depression, anxiety, post-traumatic stress, and suicidal ideation.^21^, ^22^, ^23^, ^24^, ^25^, ^26^ Early research has revealed greater levels of COVID-19 pandemic life disruptions and medical changes for dialysis and transplant patients. One recent study involving 22 adult kidney transplant candidates identified that stress resulted from the confusion and uncertainty about possible disruptions to their path to transplant.^27^ Other changes in kidney patients’ lives were decreases in transplant activity^28^; negative psychosocial impacts, such as the fear of getting infected while at dialysis^29^; and the use of telemedicine.^30^^,^^31^

During the COVID-19 pandemic, events converged that disproportionately impacted the mental health of transplant patients: isolation to avoid contracting COVID-19^7^; scarcity and limited access to their usual health-care team; fears of contracting COVID-19^7^; and an ongoing shortage of licensed mental health providers meant that many kidney patients had difficulties getting professional help to cope.^32^ Health-care providers must develop more accessible ways to stay connected with their patients and support them through major health crises full of high uncertainty and stress. For example, in July 2020, King’s College Hospital developed a free, online, web-based hub for kidney patients to manage their well-being during lockdown by providing services such as virtual workout sessions, advice videos on navigating physical isolation, and art therapy.^33^

Trying to make sense of COVID-19 and cope with the pandemic’s impact on mental health, many at-risk dialysis and transplant patients shared about how they searched through a vast amount of information, some credible and some not. They reported that there was limited information available for immunocompromised patients. Transplant patients shared about the difficulty in finding information tailored for immunocompromised patients and about the confusing information they were receiving from the news media. Our study found that patients sought information from their health-care institutions, by searching the Internet, from their friends and family members, from social media, and most commonly from new sources.

While we are still learning how health education was disseminated during the COVID-19 pandemic, our findings are consistent with research during Ebola and influenza outbreaks, when people sought health information from mass media, social media, and print media versus seeking information from hospitals and medical care providers.^34^^,^^35^ Health systems should increase their use of digital media and explore additional ways to best reach the community with health information during a crisis. Innovative solutions like the KTLRC hotline can be exceptional resources during crises when systems are trying to adapt to the new normal and disseminate accurate information as it is learned.

This study also confirmed findings from previous studies with kidney patients that found they commonly seek support from their family and faith communities to cope with the burden of CKD or dialysis and waiting for transplant.^36^, ^37^, ^38^ Faith communities have been shown to provide emotional support necessary to deal with the patient’s health condition, social support, and ways to make meaning of their daily life while living with a medical condition, many of which were shown to be helpful while social isolating.^39^

This study was exploratory and limited to those sharing challenges on a hotline during the first 9 months of the COVID-19 pandemic. Stressors continue to change as the pandemic evolves. A limitation to this study was a selection bias in individuals who self-selected by agreeing to participate in this study and those who called into the hotline. Therefore, as all participants self-selected to participate in this study, their experiences and challenges during the COVID-19 pandemic might be different from those of dialysis and transplant patients who did not participate in this study. Future research should continue to conduct larger studies of transplant patients’ levels of depression and anxiety over time, especially if waves of COVID-19 continue and COVID-19 vaccines remain less effective for transplant patients.

The pandemic created both high levels of mental health strain for dialysis and transplant patients and difficulty coordinating care by the health-care community. It provided insights into the psychological and practical challenges immunosuppressed patients face and the actions they take to protect the safety of their health, with or without a pandemic. The gaps in support services identified need to be explored so that health information is made readily available faster and addresses patients’ emotional needs.

## Supplementary Material

Supplementary File (PDF) Figure S1, Item S1.
