# Supplementary material for: Stressors and Information-Seeking by Dialysis and Transplant Patients During COVID-19, Reported on a Telephone Hotline: A Mixed-Methods Study
Source: Kidney Med. 2022 May 10;4(7):100479. doi: 10.1016/j.xkme.2022.100479 (PMC9087151; doi:10.1016/j.xkme.2022.100479)
Supplement: Supplementary File (PDF) — Figure S1, Item S1. [file mmc1.pdf]

Arevalo et al, Kidney Med, "Stressors and Information Seeking by Dialysis and Transplant Patients During COVID-19 Reported on a Telephone Hotline"

Figure S1: Themes and Subthemes

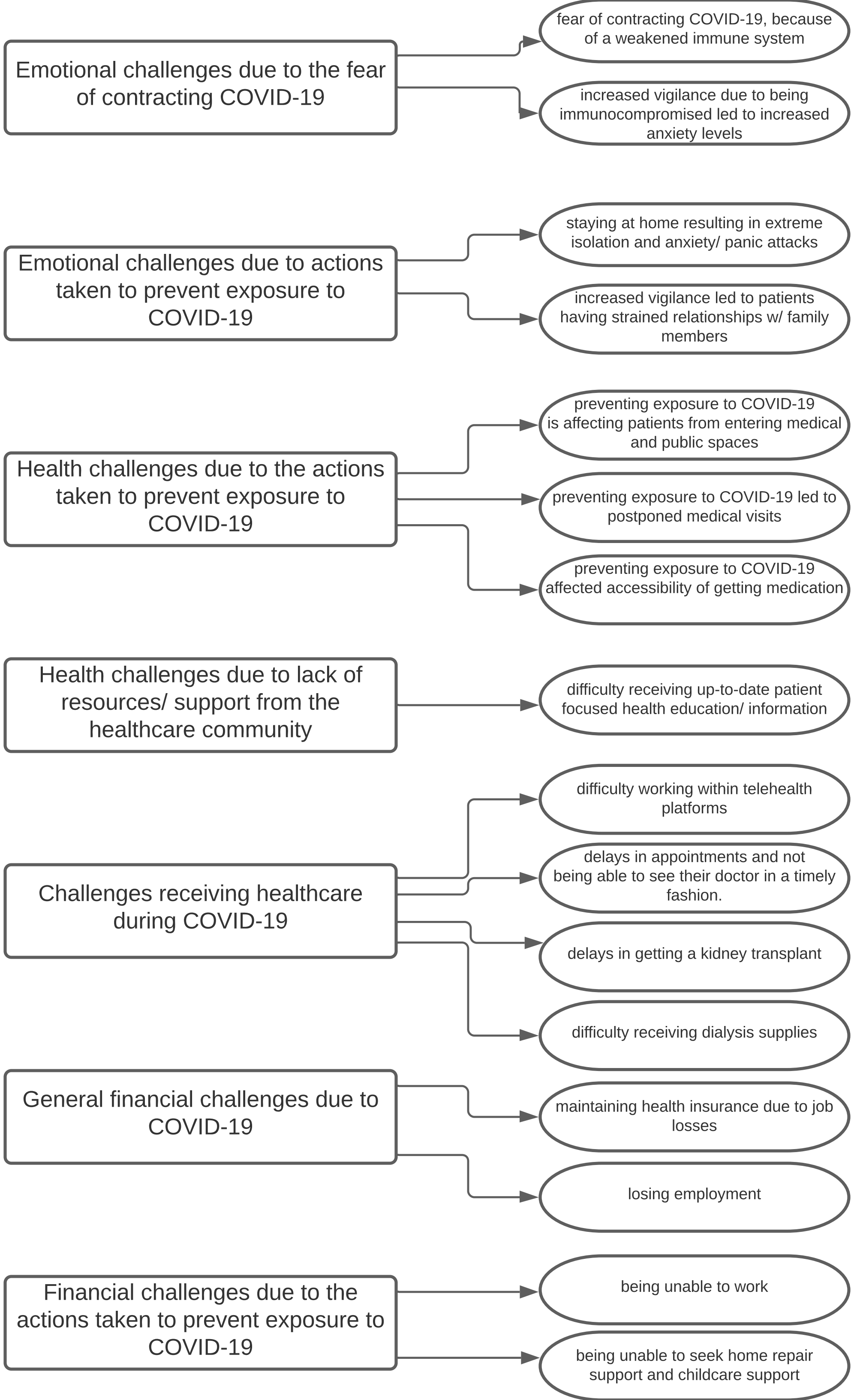

Item S1. Interview Guide

The COVID-19 Transplant Listening and Resource Center – A UCLA-TIBI Hotline
Participant Survey

STAFF: We want to hear from the dialysis and transplant community about their experiences navigating healthcare and seek information about COVID-19. We will be providing you with our current educational resources at the end of the call.

The Listening Center is part of ongoing research by the Transplant Research and Education Center at UCLA. This call will be recorded. Everything you share will be kept confidential or de-identified. Your input is extremely valuable and will help our research team to create better education for patients. Would you like to continue?

1. Are you any of these types of individuals:
   - ❑ I am a dialysis patient
   - ❑ I am a transplant patient
   - ❑ I am a caregiver for a dialysis patient
   - ❑ I am a caregiver for a transplant patient
   - ❑ I am a kidney patient (non-dialysis)

   1a. Branching logic (if Other = yes)
   If Other, please describe: _________________________

   1b. Branching logic (if I am a transplant patient=yes)
   What type of transplant did you receive (select all that apply)?
   - ❑ Kidney
   - ❑ Liver
   - ❑ Heart
   - ❑ Pancreas
   - ❑ Lung
   - ❑ Intestine, Small bowel, or Colon
   - ❑ Corneal
   - ❑ Other:______________________

   1c. Branching logic (If I am a caregiver for a transplant patient = yes)
   What type of transplant did they receive (select all that apply)?
   - ❑ Kidney
   - ❑ Liver
   - ❑ Heart
   - ❑ Pancreas
   - ❑ Lung
   - ❑ Intestine, Small bowel, or Colon
   - ❑ Corneal
   - ❑ Other:______________________

2. Has your health or the health of a family member been impacted by COVID-19? (radio button)
    - Yes
    - No

    2a. Branching (if health impacted by COVID-19= yes AND person is a tx or dialysis patient)
    Have you tested positive for COVID-19?
    - Yes
    - No

    2b. Branching (if 2a= yes AND person is a tx or dialysis patient)
    Have you been hospitalized for COVID-19?
    - Yes
    - No

    2c. Branching (if health impacted by COVID-19= yes AND person is a caregiver)
    Has the patient you care for tested positive for COVID-19?
    - Yes
    - No

    2d. Branching (if 2a= yes AND person is a caregiver)
    Has the patient you care for been hospitalized for COVID-19?
    - Yes
    - No

3a. Branching (If person is a dialysis or transplant patient)

READ: for the following two questions I am going to read a statement. Please tell me whether you strongly agree, agree, neither agree nor disagree, disagree, or strongly disagree with the statement.

How I am receiving my healthcare has changed since COVID-19. (Radio buttons)
- Strongly agree
- Agree
- Neither agree nor disagree
- Disagree
- Strongly disagree

3b. Branching (If person is a caregiver)

READ: for the following two questions I am going to read a statement. Please tell me whether you strongly agree, agree, neither agree nor disagree, disagree, or strongly disagree with the statement.

How the person I care for is receiving my healthcare has changed since COVID-19. (Radio buttons)
- Strongly agree

- Agree
- Neither agree nor disagree
- Disagree
- Strongly disagree

1. Branch (if they selected strongly agree OR agree for #3a or #3b)

Has this changed for the better or worse? (radio button)

- For the better
- For the worse

Section 2

Read Aloud: Now let's focus on your experience and any questions that you have.

2. What made you call the COVID-19 Listening and Resource Center today?

____
____
____

3. What new challenges are you or the person you take care of facing in your life since the beginning of COVID-19?

____
____
____

4. Have any of these challenges affected how you/the person you take care of? (check all that apply)

Health

- ❑ I/ the person I care for is being less physically active due to staying at home
- ❑ I/ the person I care for had a change in medication or dosing
- ❑ I have had difficulty obtaining medications
- ❑ My/the patient's dialysis center made changes to their policies
- ❑ My/the patient's dialysis center has changed due to management of COVID patients
- ❑ I/the person I care for switched to telehealth visits
- ❑ I/the person I care postponed a medical visit
- ❑ My (or my loved one's) transplant center made changes to their policies
- ❑ My transplant-related appointments were postponed
- ❑ My transplant was delayed
- ❑ My living donor was unable to donate during this time.
- ❑ Other: ______________________________

Financial

- ❑ I/ the person I care for lost my job
- ❑ My paid work hours were reduced

- ❑ I began working from home
- ❑ Other: ______________________________

Grief and Loss

- ❑ I lost a family member or friend due to COVID-19
- ❑ I am lonely due to social distancing
- ❑ I am scared about getting COVID-19
- ❑ I am frustrated about the limitations to my life due to COVID-19
- ❑ Other:__________________________________

5. What has been the most stressful for you?

____
____
____

   a. What has helped you the most?

____
____
____

   b. Is there any additional help you still need?

____
____
____
____

6. How are you seeking support at this time?

____
____
____

   a. What has helped you the most?

   ____
   ____
   ____

   b. Is there any additional help that you still need?

____
____
____

Thank you for sharing this openly with us. I am going to send you a comprehensive list of resources at the end of our call that I hope may be helpful for you.

7. What new questions have you or the patient you care for been having since the beginning of COVID-19?

____
____
____

8. PROBE: What kind of COVID-19 related questions have you had?

   ____
   ____
   ____

9. PROBE: Have you had questions about any new questions about your kidney or transplant health?

   ____
   ____
   ____

10. PROBE: Have you had new questions about how your healthcare is delivered?

   ____
   ____
   ____

11. PROBE: Are there any questions that you have had more difficulty getting answers to?

   ____
   ____
   ____

12. I know that healthcare has been changing quickly, is there anything that you think the healthcare community could do to better help you right now?

____
____
____

13. Anything else that you want to share that I didn't ask you?

____
____
____

Section 3

STAFF: Thank you so much for talking with me about your experiences! This call center is part of the Transplant Research and Education Center at UCLA. We conduct research and design

education to better serve patients, donors, and their families. Would you answer a few final questions about how you are learning about your health right now and allow us to use the information that you shared today for research purposes?

- o Yes
- o No

14. How are you learning about COVID-19 now (check all that apply):
    - ❑ From Social Media
        - ❑ Facebook
        - ❑ Private Facebook groups for patients/donors
        - ❑ Instagram
        - ❑ Twitter
        - ❑ Reddit
    - ❑ From my Healthcare Institutions:
        - ❑ My dialysis center
        - ❑ My transplant center
        - ❑ From my kidney doctor
    - ❑ From News Sources
        - ❑ Television news (printed and online)
        - ❑ Newspapers
        - ❑ Email distribution lists
    - ❑ From patient groups
    - ❑ From my friends and family members
    - ❑ By Searching the internet
    - ❑ Other: ____

15. How often have you read health information on the Internet in the past 30 days?
    - o Not at all
    - o Less than one time per week
    - o About one time per week
    - o Two or more times per week
16. How often have you read about health issues in newspapers or general magazines in the past 30 days?
    - o Not at all
    - o Less than one time per week
    - o About one time per week
    - o Two or more times per week

17. How often have you watched special health segments of television newscasts in the past 30 days?
    - o Not at all
    - o Less than one time per week

- About one time per week
- Two or more times per week

18. How often have you watched television programs (other than the news) which address health issues or focus on doctors or hospitals in the past 30 days?
    - Not at all
    - Less than one time per week
    - About one time per week
    - Two or more times per week

19. How often have you talked with family or friends about health issues in the past 30 days?
    - Not at all
    - Less than one time per week
    - About one time per week
    - Two or more times per week

20. Please rate your agreement with the following items:
    a. I wanted health information that I did not know how to get
        - Disagree very much
        - Disagree
        - Neither disagree nor agree
        - Agree
        - Agree very much

    b. I needed health information that I could not afford the time or effort to get
        - Disagree very much
        - Disagree
        - Neither disagree nor agree
        - Agree
        - Agree very much

    c. I needed health information that I could not afford to pay for
        - Disagree very much
        - Disagree
        - Neither disagree nor agree
        - Agree
        - Agree very much

    d. I am able to find good health information when I need it
        - Strongly disagree

- Disagree
- Neither disagree nor agree
- Agree
- Strongly agree

e. The amount of health information available today makes it easier for me to take care of my health
- Strongly disagree
- Disagree
- Neither disagree nor agree
- Agree
- Strongly agree

21. Which of the following types of resources would be helpful to you (check all that apply):
- ❑ Educational videos about COVID-19 and staying safe
- ❑ Website with Frequently Asked Questions answered by experts
- ❑ A live webinar for patients
- ❑ Live chat where you can ask questions to a healthcare professional

22. Do you or the person you care for have the resources needed to access care through telemedicine or mHealth (access to a computer, smartphone, or tablet)?
- Yes
- No

23. Do you have the knowledge necessary to use a telemedicine platform or mHealth app?
- Yes
- No

24. Is mobile health technology compatible with other technologies that you use?
- Yes
- No

25. Using mHealth or Telehealth services would make me very nervous
- Strongly agree
- Agree
- Neither agree nor disagree
- Disagree
- Strongly disagree

26. Using mHealth or Telehealth services make me worried.
- Strongly agree
- Agree
- Neither agree nor disagree
- Disagree

- Strongly disagree

27. Using mHealth or Telehealth services may make me feel uncomfortable.
    - Strongly agree
    - Agree
    - Neither agree nor disagree
    - Disagree
    - Strongly disagree

28. Using mHealth or Telehealth services may make me feel uneasy and confused
    - Strongly agree
    - Agree
    - Neither agree nor disagree
    - Disagree
    - Strongly disagree

29. Is English your primary language?
    - Yes
    - No

30. What is your zipcode?

____

31. How often do you find or look for health information?
    - Never
    - Not very often
    - Sometimes
    - Very often
    - Always

32. How often do you understand information about your health?
    - Never
    - Not very often
    - Sometimes
    - Very often
    - Always
33. How often do you evaluate how health information relates to your life?
    - Never
    - Not very often
    - Sometimes
    - Very often
    - Always
34. How often do you communicate about your health with others?

- Never
- Not very often
- Sometimes
- Very often
- Always

35. How often do you act on information about your health? (radio button)
    - Never
    - Not very often
    - Sometimes
    - Very often
    - Always

36. How old are you? ______ years

37. What is your gender? (select one)
    - Male
    - Female
    - Trans Male
    - Trans Female
    - Non-binary AFAB
    - Non-binary AMAB
    - Agender / Gender neutral Intersex
    - Other, please specify: _________________

38. Which of the following best describes your race? Select all that apply.
    - ❑ Black or African American
    - ❑ White
    - ❑ American Indian or Alaska Native
    - ❑ Asian
    - ❑ Native Hawaiian
    - ❑ Guamanian or Chamorro Samoan
    - ❑ Other Pacific Islander
    - ❑ Other
    - ❑ Multiracial

28a. Branching Logic. If Asian, which of the following best describes you? Select all that apply.

- ❑ Asian Indian
- ❑ Filipino
- ❑ Chinese
- ❑ Japanese
- ❑ Korean
- ❑ Vietnamese

- ❑ Other Asian

39. Are you of Hispanic, Latinx, or Spanish origin?
    - o Yes
    - o No

17a. If yes, specify:
- ❑ Mexican American, Chicano/a
- ❑ Puerto Rican
- ❑ Cuban
- ❑ Another Hispanic, Latino, or Spanish origin

40. What is the highest level of education you have completed? (radio button)
    - ❑ 8th grade or less
    - ❑ Some high school
    - ❑ High School Diploma or GED
    - ❑ Some college or vocational school
    - ❑ College or vocational school degree
    - ❑ Some professional or graduate school
    - ❑ Professional or graduate degree

41. **Branching logic**: give the following questions if they were a family member or caregiver.

How old is the dialysis or transplant patient who you care for? ____ Years.

What is the patient’s gender? (select one)
- o Male
- o Female
- o Trans Male
- o Trans Female
- o Non-binary AFAB
- o Non-binary AMAB
- o Agender / Gender neutral Intersex
- o Other

Branching logic
If gender is other, please specify: __________________

Which of the following best describes the patient’s race? Select all that apply.
- ❑ Black or African American
- ❑ White
- ❑ American Indian or Alaska Native
- ❑ Asian

- ❑ Native Hawaiian
- ❑ Guamanian or Chamorro Samoan
- ❑ Other Pacific Islander
- ❑ Other
- ❑ Multiracial

Branching Logic:

- ❑ Asian Indian
- ❑ Filipino
- ❑ Chinese
- ❑ Japanese
- ❑ Korean
- ❑ Vietnamese
- ❑ Other Asian

Is the patient of Hispanic, Latinx, or Spanish origin?

- o Yes
- o No

Branching logic

If yes, specify:

- ❑ Mexican American, Chicano/a
- ❑ Puerto Rican
- ❑ Cuban
- ❑ Another Hispanic, Latino, or Spanish origin

What is the highest level of education that the patient has completed? (radio button)

- o 8th grade or less
- o Some high school
- o High School Diploma or GED
- o Some college or vocational school
- o College or vocational school degree
- o Some professional or graduate school
- o Professional or graduate degree

42. Which of the following best describes the source of your or the person you take care of income? (Check all that apply)
    - ❑ Full-time employment
    - ❑ Part-time employment
    - ❑ Employment of others in household
    - ❑ Retirement savings/Pension
    - ❑ Social Security (Retirement)
    - ❑ Unemployment

- ❑ Welfare/TANF
- ❑ Disability due to kidney disease
- ❑ Disability due to other causes
- ❑ I prefer not to answer

43. What type of health insurance do you or the person you take care of have? (Check all that apply)
    - ❑ Medicare (National Medical Card)
    - ❑ Medicaid (State Medical Card)
    - ❑ Private Insurance (I.e. HMO, PPO)
    - ❑ Other Governmental Insurance (I.e. VA or Indian Health Service)
    - ❑ I have no insurance and pay cash for my care
    - ❑ I have no insurance and don't pay for my care
    - ❑ Other Insurance
    - ❑ Don't Know
    - ❑ I prefer not to answer

44. If your family lost your current income, how long could you continue to live in your current situation? (radio button)
    - o Less than 1 month
    - o 1-2 months
    - o 3-6 months
    - o 7-12 months
    - o More than a year
    - o I prefer not to answer

45. Do you have a washer and dryer at home?

- o Yes
- o No

46. Do you or does anyone in your household own a car or other vehicle?

- o Yes
- o No

47. Which of the following statements best describes, in general, how safe you feel in your home or neighborhood?

- o I feel very safe
- o I feel somewhat safe
- o I do not feel safe at all

48. PHQ-4: The four-item patient health questionnaire for anxiety and depression.

Over the last two weeks, how often have you been bothered by the following problems?

	Not at all	Several days	More than half the days	Nearly every day
**1.** Feeling nervous, anxious or on edge	1☐	2☐	3☐	4☐
**2.** Not being able to stop or control worrying	1☐	2☐	3☐	4☐
**3.** Feeling down, depressed, or hopeless	1☐	2☐	3☐	4☐
**4.** Little interest or pleasure in doing things	1☐	2☐	3☐	4☐

49. Is there anything else that you'd like to add?

____

____

____
